# A Pivotal Role for Mycobactin/*mbtE* in Growth and Adaptation of Mycobacterium abscessus

**DOI:** 10.1128/spectrum.02623-22

**Published:** 2022-11-02

**Authors:** Mark Foreman, Ilana Kolodkin-Gal, Daniel Barkan

**Affiliations:** a The Robert H. Smith faculty of Agriculture, Food and Environment, The Hebrew University of Jerusalem, Rehovot, Israel; Johns Hopkins University School of Medicine

**Keywords:** *Mycobacterium abscessus*, iron acquisition

## Abstract

Mycobacterium abscessus is an emerging pathogen that critically depends on iron for growth and pathogenesis. The acquisition of iron in Mycobacterium tuberculosis is governed by siderophores called mycobactins, synthesized by the *mbt* gene cluster, but the role of this gene cluster in the adaption of M. abscessus to iron limitation is not characterized. We identified an M. abscessus Tn_mutant with interruption of the *mbtE* gene (*MAB_2248c*), a central component of mycobactin biosynthesis. We tested this isolate growth characteristic, dependency on supplements, and transcriptomic response, comparing it to the response of wild-type (WT) bacteria in iron-limiting conditions. We also compare the structure of the *mbt* gene cluster across several mycobacteria. The Tn_*mbtE* mutant had a substantial, but not absolute, growth defect, which was more substantial in iron-limited media. Supplementation with mycobactin-J, hemin, blood, and surprisingly, albumin, salvaged the poor growth. Similarly, secreted mature (carboxy)-mycobactins from WT bacteria rescued the Tn_*mbtE* mutant during iron deprivation. The transcriptomic response of the Tn*_mbtE* mutant involved the upregulation of genes known to be implicated in iron homeostasis and was comparable to that of WT bacteria grown in iron-limiting conditions. Interestingly, the response was not identical to the response of M. tuberculosis to iron limitation. The *mbt* gene cluster and mycobactins play important roles in the physiology of M. abscessus. (Carboxy)-mycobactin is secreted from WT bacteria and can serve as “public good.” The role of several iron-homeostasis related genes (like *ideR*) may differ between M. abscessus and Mtb.

**IMPORTANCE** Mycobacterium abscessus is an emerging human pathogen belonging to the nontuberculous mycobacteria (NTM) family, causing severe pulmonary disease in compromised individuals. How this bacterium acquires iron is poorly understood. Here, we provide the first characterization of the role(s) the *mbtE* gene required for the biosynthesis of siderophore mycobactin in M. abscessus. We show that the gene *mbtE i*s required for growth during iron deprivation and can be compensated by several supplements, including, surprisingly, albumin. We also show the transcriptomic response of the *mbtE*-mutant is comparable to the response of the parental strain to iron starvation and seems different from the response of M. tuberculosis. These results indicate the importance of studying mycobactin in M. abscessus and NTM strains. Understanding this pathway is central to understanding the acquisition of iron within hosts and its role in pathogenesis, which in turn may facilitate the development of antimycobacterial therapeutics.

## INTRODUCTION

Iron is essential for almost all living organisms, including pathogenic bacteria. Iron levels are closely regulated, as both excess and lack of iron are detrimental. Within a host, bacteria have developed a complex mechanism to compete for iron, with many of these mechanisms being essential for full virulence. Enhancing our understanding of iron homeostasis and its contribution to bacterial survival and virulence is therefore important for developing effective treatments against many pathogens. Siderophores are small organic molecules produced by bacteria under iron-limiting conditions to enhance the uptake of iron and thus play a pivotal role in iron acquisition and pathogenesis, making them a potential therapeutic target (1).

One category of emerging pathogens is the nontuberculous mycobacteria (NTM). Within this group, Mycobacterium abscessus is one of the most clinically relevant species, with an increasing prevalence over the last 2 decades. This pathogen is associated with high rates of antimicrobial resistance and tolerance, necessitating long and complicated treatment regimens. Despite these, patients experience multiple relapses with low cure rates. A better understanding of iron metabolism could potentially enable targeting these pathways, thus adding a therapeutic option. However, in M. abscessus, and in many other nontuberculous mycobacteria, very little is known about iron acquisition and its potential role(s) in pathogenesis. The complex mycobacterial mechanisms to import iron include mycobacterium-specific siderophores called mycobactins (membrane associated) and carboxymycobactins (secreted). These are first synthesized by the bacteria and secreted by specialized secreting systems, successfully compete with host iron-binding proteins thanks to their extremely high affinity, and then are imported back (via the IrtAB transporter) into the bacterial cell. The efflux pump MmpL5 is central to mycobactin export (2), and mycobactin activity is largely dependent on the proper function of the ESX-3 secretion system and successful secretion of EsxG and EsxH, although the precise mechanism of this dependency is not fully characterized. Mycobacteria, and specifically Mycobacterium tuberculosis (Mtb) lacking the ESX-3 system, are growth defective and highly attenuated (3, 4). The synthesis of mycobactins is a complex process involving multiple enzymes and the genes coding for them, comprising the *mbtA* to *mbtJ* genes. Mycobacteria deficient in the *mbt* genes necessitate supplementation for growth *in vitro* either in the form of a functional mycobactin (like mycobactin-J for M. avium*-paratuberculosis*) or hemin (like for M. haemophilum), bypassing the need for mycobactins altogether. *In vivo*, mycobacteria can also utilize heme as an iron source (5, 6), but for most mycobacteria, proper mycobactin synthesis, export, and then import are essential for full pathophysiology. For example, a Bacille Calmette-Guérin mutant defective in *mbtB* was loaded with iron during cultivation and then tested as a vaccine intended for highly immune-suppressed patients. These bacteria could not persist beyond several replication cycles even in severe combined immunodeficiency mice. Whereas a relative lack of iron may signal the bacteria to increase its pathogenic properties (such as persistence and antibiotic tolerance in Mtb), complete lack of iron (or inability to acquire it) is highly detrimental to the bacteria.

In this study, we provide a first in-depth characterization of an M. abscessus *mbtE*-inactivation mutant. *mbtE* is one of the central mycobactin-synthesis-related genes. Mtb lacking *mbtE* is growth defective *in vitro* and attenuated in macrophages and guinea pigs (7). We used an *mbtE* (*Mabs_2248c*) transposon mutant to characterize some of the phenotypes associated with *mbtE* disruption in M. abscessus. We show this mutant is, although viable, significantly defective in growth. The growth defect could be ameliorated by mycobactin-J and hemin, as well as by the supplement oADC (containing bovine albumin), but not by forms of inorganic iron. The defect was specific to the functional deletion of *mbtE*, and caused by a lack of a secreted component (namely, mature mycobactin). Furthermore, our results indicate that *mbtE* products may serve as a “public good” as the WT secretome can compensate for the loss of *mbtE.* We also characterized the transcriptomic response to the *mbtE* defect and showed it to resemble the response to iron-limiting conditions. Collectively, our results demonstrate the primary function of mycobactin to supply soluble iron from multiple sources to M. abscessus during undisturbed growth as well as under iron limitation.

## RESULTS

### Isolation of an *mbtE* transposon mutant.

**FIG 1:**
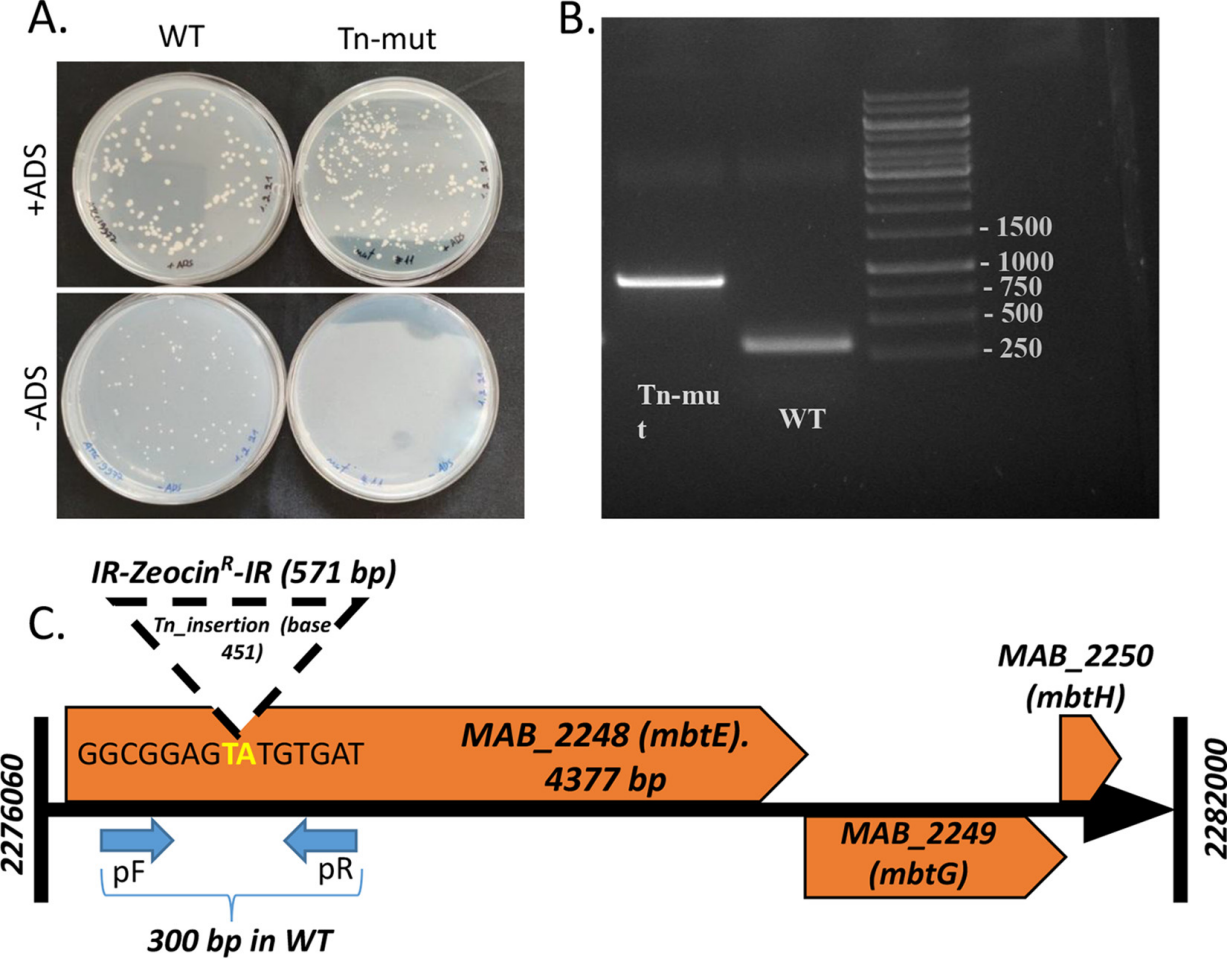
Isolation and identification of an *mbtE* Tn-mutant. (A) A Tn-mutant growing normally on 7H9/agar/glycerol/ADS plates (top panel) but not on plates without ADS (bottom panel) was identified. (B) A targeted PCR amplifying a 300 bp fragment of *mbtE* in WT bacteria was performed (see panel C for schematic representation). In the Tn-mutant, an 871 bp fragment resulted from the PCR. The 871-bp fragment was also sent for Sanger sequencing, confirming the Tn-insertion site.

Using a recently created and described M. abscessus *ATCC_19977* transposon-mutant library, we screened for Tn-mutants with a growth defect on 7H9/glycerol agar media without ADS (albumin-dextrose-NaCl), compared to similar media with ADS. We isolated one such mutant (Fig. 1A) and identified the Tn-insertion site. This was found to be the TA dinucleotide at position 451 in the gene *Mabs_2248c*. The insertion position was verified using a directed PCR that produces a 300-bp fragment in WT and an 870-bp fragment (300 + 570 bp of *zeo^R^*) in the transposon mutant (Fig. 1B and C). *Mabs_2248c* is a 4,377-bp gene coding for a 1,458 amino acid (aa) protein; the Tn insertion at position 451 truncates the protein after 150 aa, effectively producing a deletion mutant. The full-length protein is a homolog of Mtb *Rv_2380c* (*mbtE*) (51% homology), which was shown to be essential for normal growth and virulence in Mtb (7) but is still uncharacterized in NTM. We therefore, opted to use this Tn-mutant (named mDB274) to study the role of *mbtE* in M. abscessus.

### Tn_*mbtE* has a severe growth defect ameliorated by several, but not all, forms of iron as well as by mycobactin-J.

**FIG 2:**
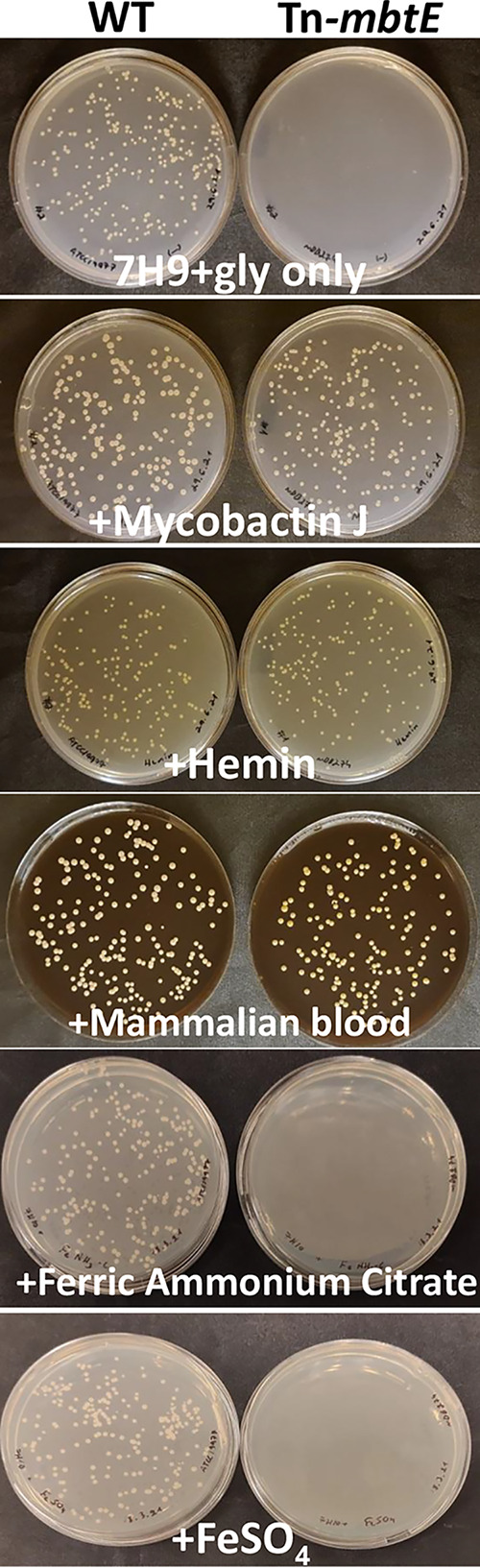
When plated on 7H9/glycerol agar plates, the Tn_*mbtE* mutant could be salvaged by mycobactin-J, hemin, and blood but not inorganic iron. Approximately 250 CFU were plated on each plate, and kept at 37°C for 5 days (WT and the mutant when compensated by mycobactin-J (MJ), hemin, and blood) and up to 10 days for the Tn mutant when compensation by inorganic iron was attempted (still with no apparent growth).

In Mtb, *mbtE* is important for iron acquisition through its central role in mycobactin (siderophore) synthesis. We sought to establish if supplementation of the growth media by excess iron in various forms could salvage growth. We plated equal amounts of WT and Tn-*mbtE* bacteria (~250 CFU on 7H9/glycerol/agar plates), supplemented by either mycobactin-J, hemin (1.5% vol/vol of a 2 mM solution), mammalian blood (10%), 1.5 mM FeSO_4_ (Hepta-hydrate) and 60 μM ferric ammonium Citrate {(NH_4_)_5_[Fe(C_6_H_4_O_7_)_2_]}. Whereas mycobactin-J, hemin, and blood salvaged the defective growth phenotype, neither FeSO_4_ nor (NH_4_)_5_[Fe(C_6_H_4_O_7_)_2_] did the same (Fig. 2). Notably, mycobactin-J could compensate for the loss of *mbtE* in M. abscessus, indicating a permissive recognition by mycobactin update systems.

**FIG 3:**
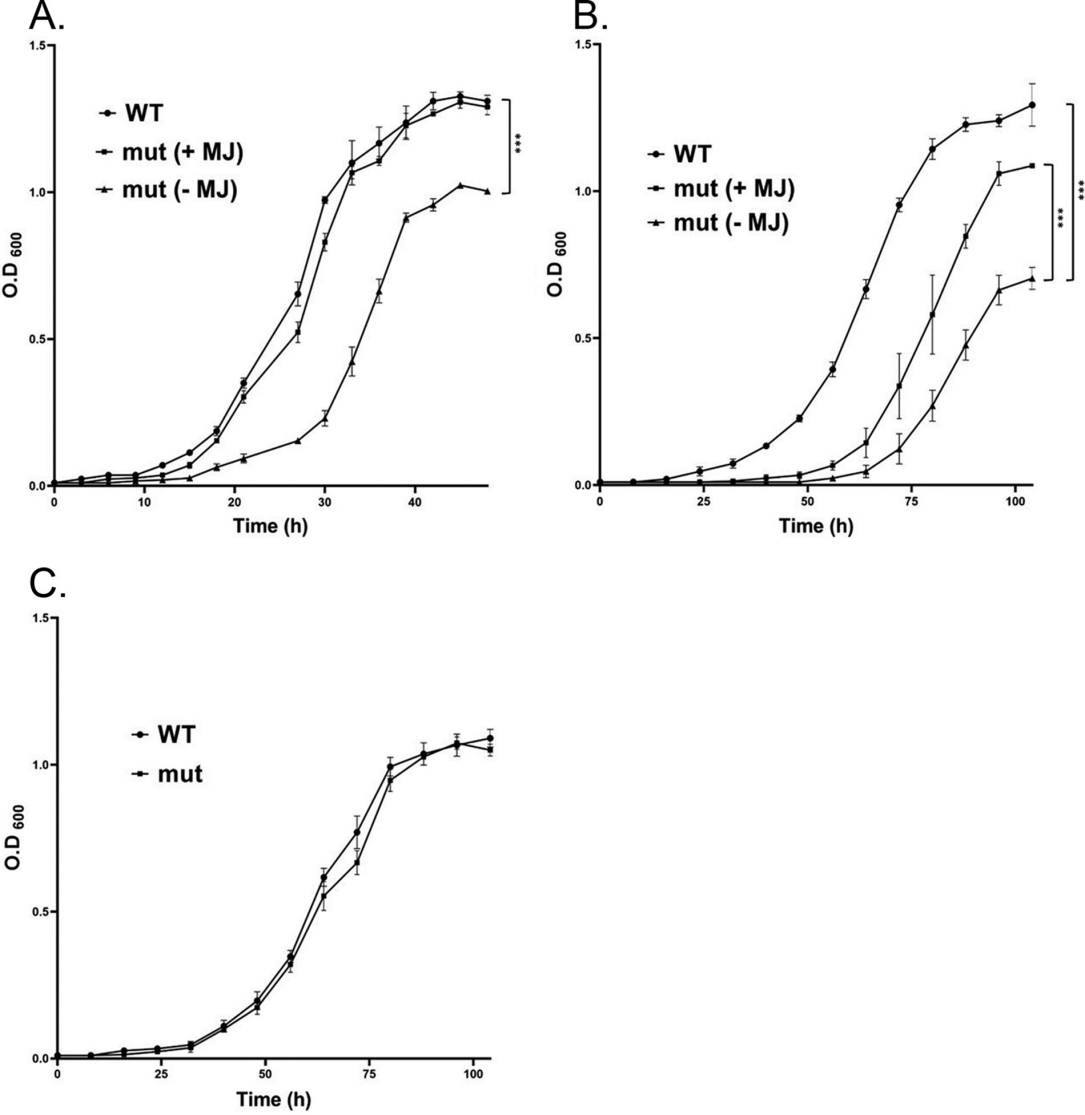
Mycobactin-J rescues the slow-growth phenotype of the Tn_*mbtE* mutant. (A) When grown in 7H9+glycerol, the Tn_*mbtE* mutant has only a slight growth retardation phenotype (Doubling times: WT, 5 h; Δ*mbtE*, 6.4 h; Δ*mbtE* + MJ, 5 h). ***, *P* < 0.001. (B) When first grown in iron-depleted Sauton’s media for 10 days, and then transferred to 7H9/glycerol, the growth retardation phenotype is more evident, and mostly compensated by MJ (doubling times: WT, 10.2 h; Tn_*mbtE*, 15.9 h; Tn_*mbtE* + MJ, 10.6 h). ***, *P* < 0.001. (C) When first grown in Sauton’s media for 10 days, and then transferred to 7H9/glycerol media previously “used” by WT bacteria, the growth defect of the Tn_*mbtE* mutant is completely reversed, as in addition of MJ. All experiments were performed in triplicates. A representative experiment out of two is shown. OD_600_, optical density at 600 nm.

The Tn-*mbtE* mutant was isolated by observing a growth defect on 7H9/glycerol agar plates. We, therefore, tested the growth curve of the mutant compared to WT in 7H9/glycerol liquid media. We first noticed a prolonged resuscitation phase, with an almost normal logarithmic growth afterward (Fig. 3A). This was seen in bacteria grown in 7H9/glycerol (without ADS), seeded from media with ADS. The addition of mycobactin-J seemed to revert the growth to near normal, but as the preliminary defect was subtle, so was the effect of mycobactin-J. However, when bacteria (WT and mutant) were first grown in the relatively iron-poor Sauton’s media for 10 days, and only then seeded into the experiment tubes (with 7H9/glycerol) for growth measurement, the mutant showed a defect in the logarithmic phase as well (Fig. 3B). Most of the growth defect could be reverted by adding mycobactin-J to the media, suggesting that the acquisition of iron, especially from the relatively iron-poor medium was the main defect in the Tn-*mbtE* bacteria. Interestingly, the growth defect was also relieved by growth in media with ADS (even compared to the same media without ADS). This could be due to iron acquisition through endocytosis of iron bound to albumin.

To show that the growth defect is due to a lack of a secreted, soluble iron chelator (mycobactin and/or carboxymycobactin) and not the ability to import or use mycobactin-bound iron, we first preincubated WT and mutant bacteria in iron-poor conditions (Sauton’s media) for 10 days and then seeded them into “used” 7H9/glycerol media (that was used for 5 days to grow WT bacteria and then filter sterilized). In contrast to the previous experiment, where “iron-starved” mutant bacteria had severe growth defects in the logarithmic phase, this defect completely disappeared when grown in used media (Fig. 3C), suggesting a soluble factor in used media could ameliorate the defect. These results indicate that mycobactin (and probably carboxymycobactins, as these are secreted whereas mycobactins are membrane associated) serves as a public good and can be taken by cells that do not produce it.

**FIG 4:**
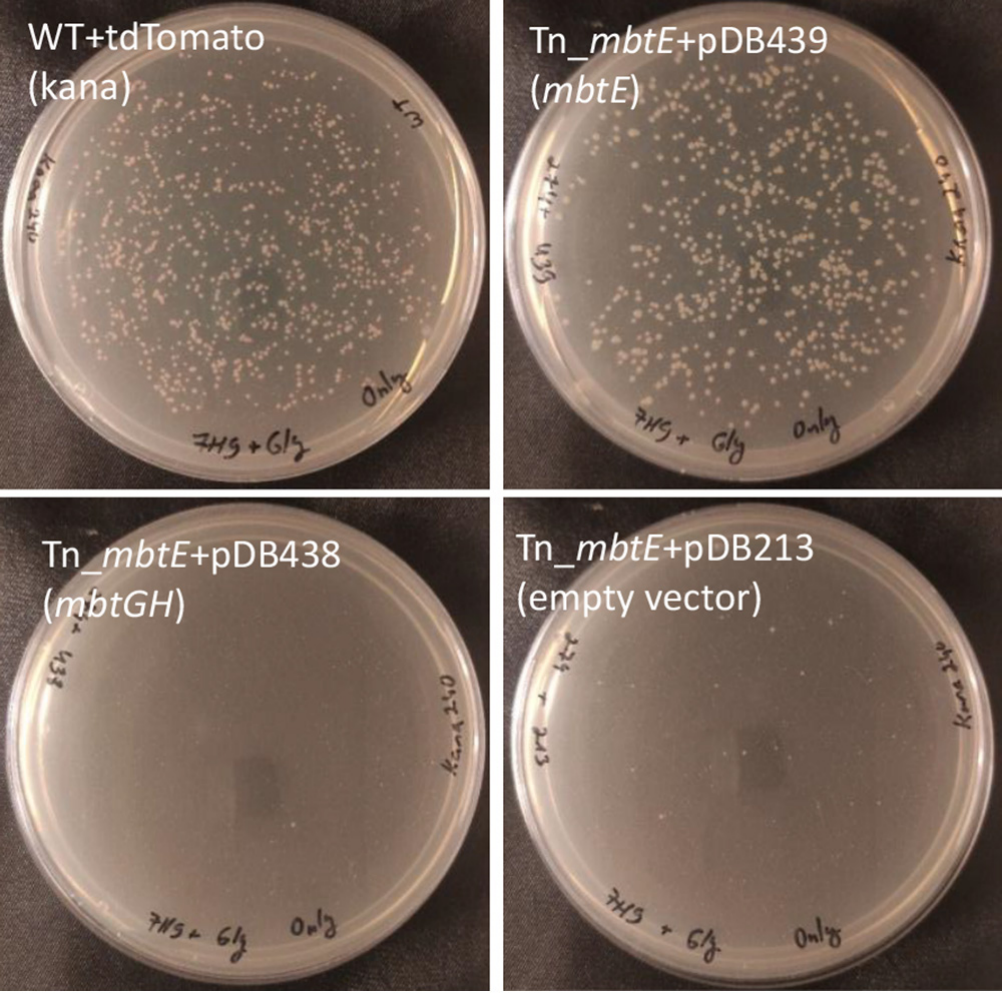
*mbtE* (top right), but not *mbtGH* (bottom left), rescue the growth defect phenotype of the *Tn_mbtE* transposon mutant. All strains were plated on 7H9/glycerol agar plates, and a wild-type mutant with a fluorescent kanamycin-selected plasmid was used as control (top left).

To confirm the specificity of the transposon effect to *mbtE*, we performed complementation experiments. The *mbtG* and *mbtH* genes are located immediately downstream of *mbtE*. Transposon insertion in *mbtE* could therefore potentially interfere with *mbtG* and *mbtH* expression, and the phenotype could be due to the combined absence (or even just that of *mbtG/mbtH*). We, therefore, cloned the full *mbtE* gene into an *attB* integrating plasmid (pDB439). We also cloned the *mbtGH* genes (with a constitutive promoter, as they could all be part of one operon driven by the *mbtE* upstream promoter, and not having an independent one) into a similar vector, creating pDB438. We complemented the Tn_*mbtE* mutant by either *mbtE* (pDB439), the combined *mbtGH* construct (pDB438), or an empty vector (pDB213). Whereas complementation with *mbtE* restored the ability of the bacteria to grow on 7H9/glycerol/agar plates, the electroporation of pDB438 (*mbtGH*) or pDB213 (empty vector) did not revert the bacteria to WT phenotype (Fig. 4). We, therefore, conclude that the absence of *mbtE* is the direct cause of the observed growth defect.

### *mbtE* disruption causes a transcriptome-adaptive response.

**FIG 5:**
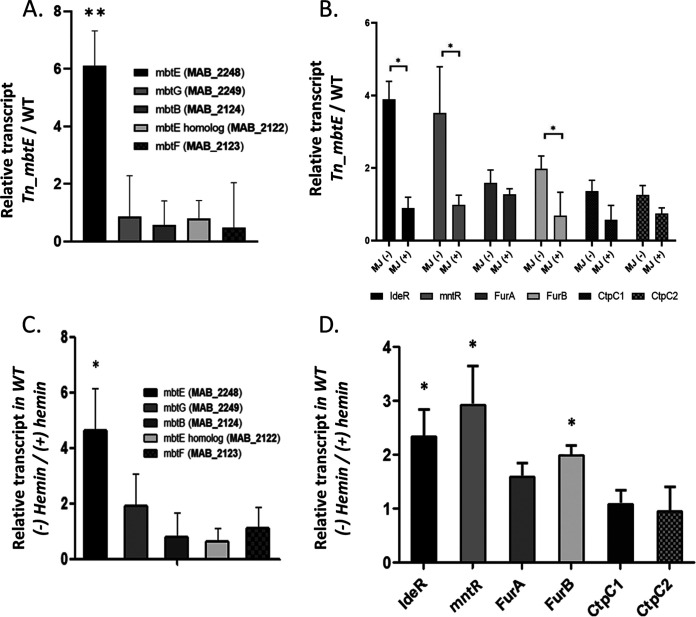
Transcriptomic response to *mbtE* inactivation is very similar to that of iron limitation. (A) *mbtE* (*MAB_2248c*, the part upstream to the transposon) is upregulated X6 in the *Tn_2248c* mutant relative to WT, whereas *MAB_2122c* is virtually unchanged. *P* = 0.001. (B) Additional, but not all, genes related to iron homeostasis are upregulated in the *Tn_2248c* mutant, and this upregulation is not present when the mutant is supplemented by mycobactin-J (MJ). *, 0.01 < *P* < 0.05. (C and D) The response of WT bacteria, when grown in iron-limiting conditions (Sauton’s media, +/− hemin), is very similar to that of the *Tn_2248c* mutant. *P* = 0.011 (*mbtE*), 0.019 (*ideR*), 0.038 (*mntR*), and 0.041 (*furB*). All experiments were performed three independent times (biological replicates), each in triplicates (technical replications). The Wilcoxon signed-rank test was used.

The prior experiments suggested that *mbtE* absence is deleterious for growth in iron-poor conditions, as the availability of iron to the bacteria is reduced due to a lack of soluble siderophores. We, therefore, hypothesized that the loss of *mbtE* may lead to iron starvation, which is associated with an increased expression of iron uptake genes. Using reverse transcription-quantitative PCR (qRT-PCR), we examined the effect of *mbtE* interruption on the expression of several genes, including *mbtE* itself. The transcription of *mbtE* (measured by primers amplifying fragments upstream to the transposon insertion) was upregulated 6.1 times over the WT and returned to the WT levels when mycobactin-J was added to the growth media. In contrast, no significant change was seen in the transcription of *mbtG* and *mbtH* (Fig. 5A).

*MAB_2122* is a 5,028-bp gene, which is designated in some databases as *mbtE* (in addition to *MAB_2248*, *mbtE*, which is the focus of our study). MAB_2122 protein carries very low identity (30.3%) and similarity (42.8%) to MAB_2248 protein, but it does carry similarity to Mtb’s MbtE (55%, almost similar to that of MAB_2248). Downstream of *MAB_2122* are two genes designated *mbtF* and *mbtB*. However, in our Tn-*mbtE* mutant, no upregulation in the transcription of *MAB_2122*, *mbtF*, or *mbtB* was observed (Fig. 5A). No upregulation of these genes was noted in WT bacteria under iron-limiting conditions either (Fig. 5C).

*MAB_3029* and *MAB_3110* are homologs of the Mtb *ideR* and *mntR* (also called *sirR*), respectively. Mtb *ideR* (*Rv_2711*) is a well-studied iron-responsive transcription factor. Mtb *mntR* (*Rv_2788*) is also a transcriptional regulator, involved in iron and manganese homeostasis. Due to its role in Mn^2+^ homeostasis, the name *mntR* was suggested (8), replacing *sirR*. Transcription levels of both *MAB_3029* and *MAB_3110* were upregulated in the Tn_*mbtE* mutant X3.9 and 3.5, respectively, and returned to normal upon the addition of mycobactin-J to the growth media (Fig. 5B). Other M. abscessus homologs of Mtb genes known to be involved in iron homeostasis were upregulated in a more subtle way (*Mab_2471c*, *furA*; *MAB_1678c*, *furB*; *MAB_0449*, *ctpC1*; *MAB_4853*, *ctpC2*), and this was also reversed in the presence on mycobactin-J (Fig. 5B).

We compared the changes in the transcription profile of these genes in the Tn_*mbtE* mutant to the changes in their transcription profile in WT M. abscessus, when grown in relatively iron-poor or iron-rich conditions. For iron-poor media, we used Sauton’s media, which is not completely devoid of iron but contains roughly 20% iron compared to Middlebrook 7H9. We grew WT Mabs ATCC 19977 in Sauton’s media for 10 days and then split the culture into similar Sauton’s media or Sauton’s media supplemented by 1.5% hemin (thus eliminating iron-limiting conditions while otherwise staying in similar media). After 24 h, bacteria were collected and the transcription profile of the iron-homeostasis genes tested previously was again measured. We found the transcription profile of WT Mabs grown in iron-limiting [(−)hemin] conditions to mimic that of the Tn_*mbtE* mutant (Fig. 5C and D), suggesting intracellular iron-limiting conditions are present in both situations, driving a similar transcriptomic adaptive response.

### Heme and hemin toxicity in M. abscessus.

**FIG 6:**
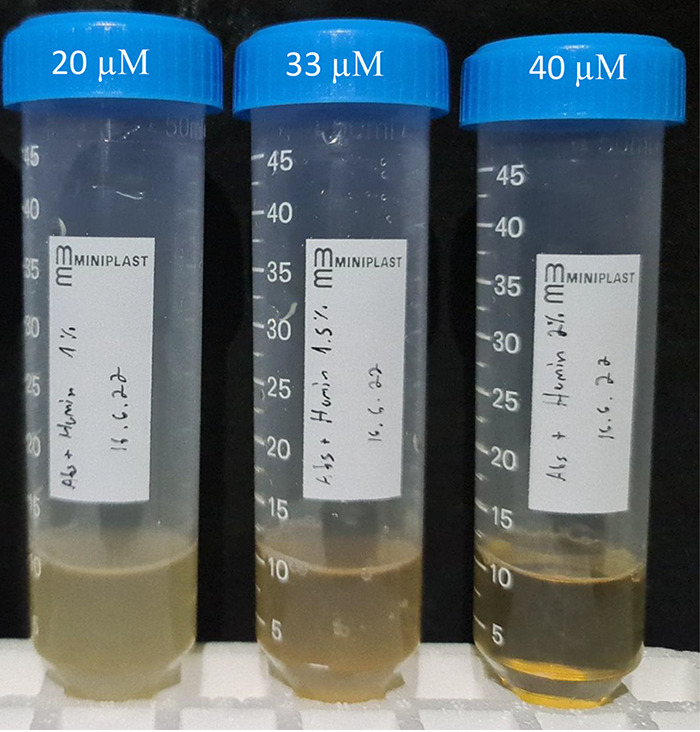
Hemin toxicity in M. abscessus. M. abscessus ATCC 19977 was grown in 7H9/glycerol with 1, 1.5, and 2% concentrations of a 2-mM stock solution of hemin, corresponding to 20-, 33-, and 40-μM concentrations. One out of three independent experiments, each with *de novo* preparation of the media and all with similar results, is shown.

A related question is whether heme poisoning exists in M. abscessus, as it does in some other bacteria. Staphylococcus aureus was found to be strongly inhibited by hemin in concentrations as low as 10 μM (9), whereas M. tuberculosis grew well in concentrations of 100 μM (3). The question of hemin poisoning was discussed in (2), with the authors suggesting Mtb was relatively resistant to heme poisoning. We performed a simple MIC to hemin experiment with wt M. abscessus. While M. abscessus grew normally at a 1.5% concentration of our stock solution (corresponding to 33 μM), growth was completely abolished at a 2% solution (40 μM) (Fig. 6). Of note, in solid media, we used concentrations as high as 66 μM, with no apparent growth retardation, suggesting a slightly different toxicity profile.

### A comparison of *mbt* genes across mycobacterial species.

**FIG 7:**
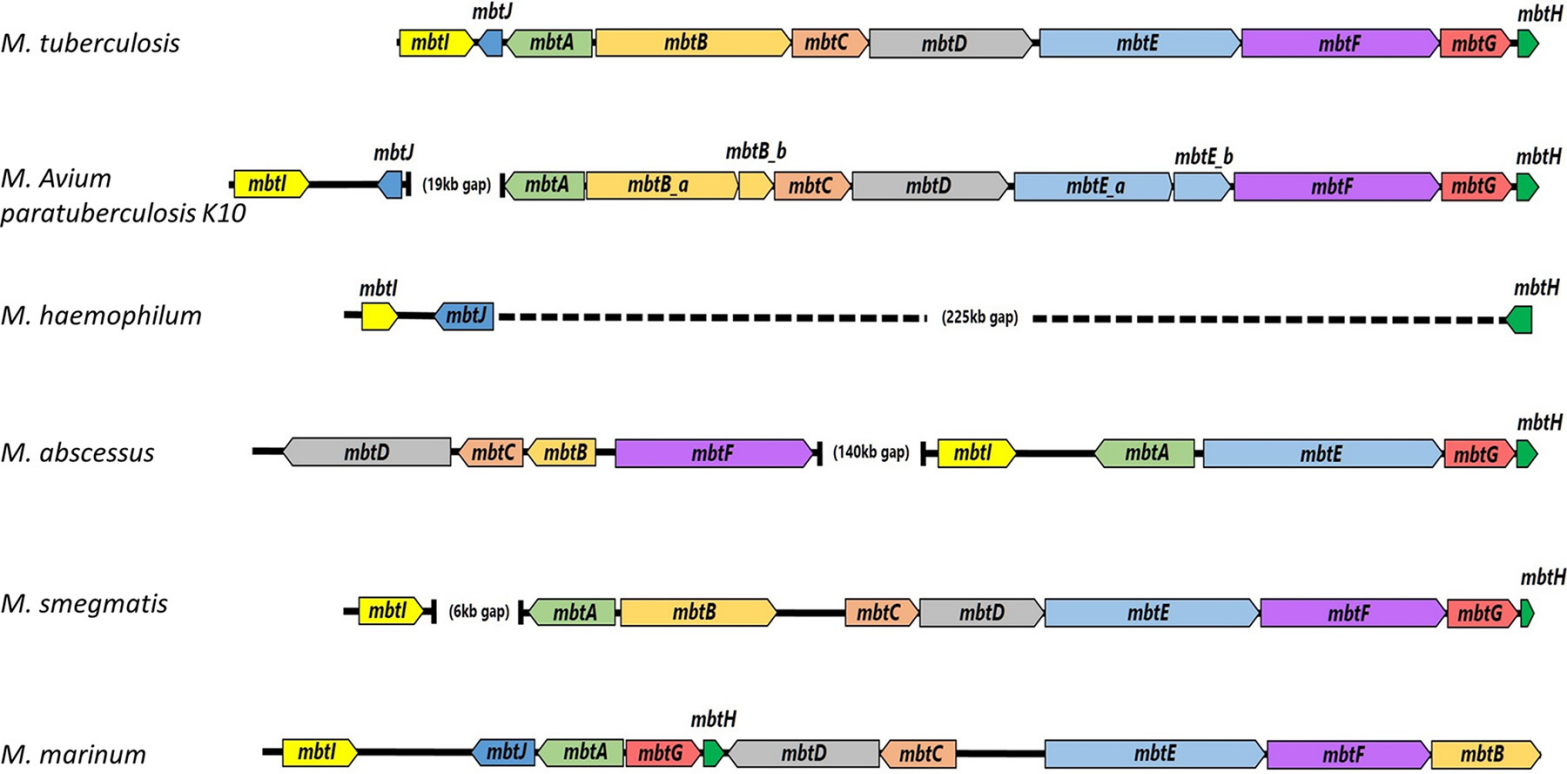
Schematic representation of the *mbt* genes organization in several important mycobacteria. Whereas in Mtb the genes are tightly packed together, in other mycobacteria small (*like* M. marinum, M. smegmatis) and large (M. abscessus) gaps are present. Large (M. haemophilum) and small (M. avium *paratuberculosis*) deletions exist in some, necessitating supplementation when grown *in vitro*.

The *mbt* genes exist across most of the mycobacteria, with the notable exception of M. haemophilum, which lacks many of them, indeed requiring iron supplementation *in vitro*. We summarized (Table 1) the *mbt* genes from several important mycobacteria, and calculated their percent identity to the genes of M. tuberculosis. In Fig. 7, we present the genomic loci organization of these genes in several mycobacteria.

**TABLE 1:** The percent identity in amino acids between the designated Mbt proteins in several mycobacteria and their respective homologs in M. tuberculosis *H37Rv*

Species	MbtA	MbtB	MbtC	MbtD	MbtE	MbtF	MbtG	MbtH	MbtI	MbtJ
M. smegmatis	71%	62%	78%	50%	61%	62%	77%	76%	68%	31%
M. abscessus	64%	51%	50%	38%	51%	42%	71%	75%	65%	48%
*MAP k-10*	78%	71%	72%	46%	66%	61%	88%	79%	74%	68%
M. marinum	72%	51%	52%	38%	44%	42%	67%	76%	79%	76%
M. haemophilum								71%	88%	51%

## DISCUSSION

Iron is essential for the pathogenesis of most bacteria, including Mycobacterium tuberculosis. Although one would presume much is shared in iron metabolism and requirements between M. tuberculosis and the emerging pathogen M. abscessus, this was not experimentally tested. Also, as some pathogens from the mycobacteria family differ in their iron metabolism from the “mainstream” paradigm of Mtb (such as M. avium*-paratuberculosis* and M. haemophilum), *a priori* assumptions regarding other mycobacteria may prove wrong and should be experimentally tested. In this study, we used an M. abscessus *mbtE* transposon mutant (effectively an *mbtE*-null isolate) to study the role of *mbtE* in M. abscessus viability, the response to iron-limiting conditions, the ability to use an alternative iron source (hemin) and the phenomenon of hemin toxicity.

We found that, much like in Mtb, an *mbtE*-defective mutant is growth retarded, although to a lesser extent than the *ΔmbtE*-Mtb mutant described previously (7). This growth defect was shown to be related to the lack of a secreted component, as media from WT bacteria could salvage the phenotype of the Tn_*mbtE* mutants. In addition to hemin and mycobactin-J, which also salvaged the phenotype, supplementation by ADS (containing albumin) also salvaged the phenotype, presumably due to iron bound to albumin. Interestingly, oADC did not (or only partially) salvage the Mtb *ΔmbtE* mutant described in reference 7. As Mtb grows poorly without oADC, it would be difficult to examine if the residual slow growth observed in that study was thanks to partial salvage by the albumin component. However, this may provide a partial answer to the question raised by other researchers, as to the reason why *mbt* mutants do not come up in transposon-library essentiality experiments (2): the explanation provided by the authors there assumed these mutants are “compensated” by the secretion of mycobactins and carboxymycobactins by other bacteria in the library, indicating that mycobactins is a “public good” that can be shared among cells but also be exploited by cheating mutants (a perfectly feasible explanation). However, an additional explanation could be partial salvage by the albumin component in the growth media.

We also show that in response to relatively iron-limiting conditions (Sauton’s media without ADS), the transcriptomic response is an increase in the transcription of some (but not all) *mbt* genes and that this response is probably due to lack of intracellular iron (as the Tn_*mbtE* mutant has a similar response). Interestingly, the iron-responsive regulator *ideR* is increased in both the Tn_*mbtE* mutants and in a WT strain grown in Sauton’s media. Previous reports explored the transcriptomic response of Mtb to iron limitation (2, 10), but we did not find a report on the specific response of the *ideR* transcript. Another study examined the effect of *ideR* deletion on the transcription of iron-related genes (11). As *ideR* was found to be essential, no direct experiments could be made. However, *ideR* was found to be transcribed in high-iron conditions and represses *mbt* transcription. This is in contrast to our results, where *ideR* was upregulated both in iron-limiting conditions and in the *mbtE* mutant. One should also note that *ΔideR* mutants were created in M. smegmatis and that the failure to produce a full *ΔideR* mutant in Mtb may in fact be due to a slow growth phenotype, rather than to true absolute essentiality (as was shown for other genes where prolonged incubations produced deletion mutants of genes thought to be essential [12]). All this suggests that the role of *ideR* in Mtb may have to be revisited.

The role of the gene *MAB_2122c* (also designated *mbtE* in databases) and the two genes immediately after it (*MAB_2123c mbtF* and *MAB_2124 mbtB*) warrant attention. It appears MAB_2122 is as similar to Mtb’s MbtE (55%) as is MAB_2248 (52%). There is no other homolog in Mtb. The similarity between MAB_2122 and MAB_2248 is only 42.8%. It appears, therefore, that although the two proteins are not very similar, they are both homologs of Mtb’s MbtE. One could therefore postulate that they are partially (or fully) redundant. However, it is clear that the inactivation of MAB_2248 (in this present work) is not compensated (at least not to a significant degree) by MAB_2122. Also, iron-limiting conditions lead to an increase in the transcript of *MAB_2248c* (~X5), but have no effect on the transcription of *MAB_2122c* (Fig. 5). It is possible that this is a gene duplication of some kind, where either the function of MAB_2122 or its regulation (or both) were somehow affected (probably in a detrimental way). The creation of a targeted MAB_2122c deletion mutant, and probably a double MAB_2122c/2248c mutant, would shed more light on this question.

We also show the genomic organization of the *mbt* genes in various mycobacteria. Indeed, heme (and related forms of iron)-dependent mycobacteria such at M. haemophilum have a deletion of most of these genes, whereas in M. avium *paratuberculosis* (MAP) the gene *mbtJ* is truncated, and additional *mbt* mutations make the bacteria dependent on alternative, less efficient iron acquisition pathways (probably related to heme) (13). The addition of mycobactin-J to MAP cultures is not an absolute requirement, but it does substantially promote growth for this difficult-to-cultivate organism, indicating a central role for siderophores secretion and uptake in mycobacteria.

In summary, we show that much like in Mtb, the *mbt* pathway is important, but not essential, for normal growth of M. abscessus. Further characterization of iron metabolism and homeostasis in M. abscessus is needed, as are studies on its role in pathogenesis. Furthermore, the central role of *mbt* pathway highlights siderophore-targeting or siderophore-based compounds as a fruitful route to combat NTM infections.

## MATERIALS AND METHODS

### Bacterial strains, mutant construction, and growth conditions.

The M. abscessus was the “standard” ATCC 19977, smooth colony-morphology strain. The creation of the transposon mutant library was performed as previously described (14), on 7H9/agar plates supplemented with 10% oADC (or ADS), 0.05% glycerol, and 50 μg/mL of zeocin for positive selection. Identification of the Tn-insertion point was done as described in reference 14. For a PCR confirming the insertion point (Fig. 1B and C), we used primers listed in Table S1 in the supplemental material. The 871-bp PCR fragment originating from the Tn_mutant was also sent for Sanger sequencing for unambiguous identification.

For complementation, we cloned *mbtE* with its native promoter or mbtGH with a constitutive promoter (as the native promoter may have been that of *mbtE*, over 4,500 bp upstream) into pDB213, a kanamycin-selected, *attB*-integrating plasmid. For selection in M. abscessus, we used kanamycin 240 μg/mL.

For growth rate measurements, we used liquid media of 7H9/0.05% glycerol/Tween80 without oADC. For iron-poor media, we used Sauton’s media/0.05% glycerol/Tween80. Prism version 8 was used for growth curve analysis.

For supplementation with hemin, a 2-mM stock solution was prepared, and the growth media or plates were supplemented by 1.5% vol/vol of this solution. For hemin toxicity experiments, an “MIC” of this solution was prepared with concentrations of 0.5, 1, 1.5, 2, 2.5, and 3%. The experiment was carried out three independent times.

### qRT-PCR.

RNA was extracted from log-phase cultures using the mirVana kit (Invitrogen). cDNA was prepared using the High Capacity cDNA Reverse Transcription kit (catalog number 4368813; Applied Biosystems). The primers used for the qRT-PCR are listed in Table S1.

### Data availability.

All data are presented in the manuscript. Additional data, as well as materials, may be requested directly from the authors.
