# Supplementary material for: A Pivotal Role for Mycobactin/mbtE in Growth and Adaptation of Mycobacterium abscessus
Source: Microbiol Spectr. 2022 Nov 2;10(6):e02623-22. doi: 10.1128/spectrum.02623-22 (PMC9769883; doi:10.1128/spectrum.02623-22)
Supplement: Supplemental file 1 — Table S1. Download spectrum.02623-22-s0001.pdf, PDF file, 0.3 MB [file spectrum.02623-22-s0001.pdf]

For rtPCR - Sequence	
5 – GTGCATGTCAAACCCAG -3	*16S*
5 – GGGATCCGTGCCGTAGC - 3	
5 - TGCGGCCGGCGAAGTTGA -3	*mbtB*
5 - ATCAAGACCCGGTAGCTC -3	
5 - TCGCCGAGTTTCGACGTG -3	*mbtE*
5 - TCACTCCCGGAAGTGACA -3	
5 - GACAATTCGTGGGAGGTC -3	*MAB_2122*
5 - AATACGTGCTCGCGGCTC -3	
5 - ACATCGCCTGGCTCAACG -3	*mbtF*
5 - TTGCCGAAGCCGTGCGGT -3	
5 - CGCGTTGCCGTGATCG -3	*mbtG*
5 - TCCGGTTCAGCGCGTC -3	
5 - TCGGCGACAACCATCAC -3	*furA*
5 - GGCACAGACCCCAGTAG -3	
5 - TGCGTTCCACCCGTCAG -3 -3	*furB*
5 - GCAGCACATCCACCTCAC -3	
5 - ATCCGTAGCCGCACAC -3	*ctpc1*
5 - GCCAAAGACCGCATCTC -3	
5 - CCGAACAATCCCGCAAG -3	*ctpc2*
5 - GCACCGAAATCGCCAG -3	
5 - GTTGCCGTGGGAGGAC -3	*ideR*
5 - CGCTGGCACTCTCGGA -3	
5 - GAGCACCGCATCTGAATC -3	*sirR*
5 - TTCATCCCAGCCATAGCC -3	

**For complementation** - **Sequence**	
5 - AGCAGCGATTCGACGGTGTTGCCCG -3	*mbtE*
5 - AATCACCATTGCTCCTTTGCCAGAATT -3	
5 - GATCCGATAACACAGGAACAGATTGTGACGTCGACATTGGCAGTGATAG -3	*mbtG* (forward)
5 - TCCCTATCCCGCCATCACGTCGCGCAGGCT -3	*mbtH* (reverse)
5 - ATCCGATCCAGACCCCAGGCTTGAC -3	MOP promoter (forward)
**Tn insertion varification**	
5 – AGCACGATGTGACCGACCTT - 3	300 bp in WT, 871 bp in *mbtE* Tn-mutant
5 – TCCTCGCGCCAATAGTCCAC - 3	

Supplementary Table 1: primers used in this study for qRT-PCR, complementation and verification of the Tn-insertion point in the Tn_*mbtE* mutant.
